# Environmental Factors Regulate Plant Secondary Metabolites

**DOI:** 10.3390/plants12030447

**Published:** 2023-01-18

**Authors:** Mirwais M. Qaderi, Ashley B. Martel, Courtney A. Strugnell

**Affiliations:** 1 Department of Biology, Mount Saint Vincent University, 166 Bedford Highway, Halifax, NS B3M 2J6, Canada; 2 Department of Biology, Saint Mary’s University, 923 Robie Street, Halifax, NS B3H 3C3, Canada

**Keywords:** abiotic stress, climate change, drought stress, elevated carbon dioxide, higher temperature, light quantity and quality, secondary metabolites, ultraviolet-B radiation

## Abstract

Abiotic environmental stresses can alter plant metabolism, leading to inhibition or promotion of secondary metabolites. Although the crucial roles of these compounds in plant acclimation and defense are well known, their response to climate change is poorly understood. As the effects of climate change have been increasing, their regulatory aspects on plant secondary metabolism becomes increasingly important. Effects of individual climate change components, including high temperature, elevated carbon dioxide, drought stress, enhanced ultraviolet-B radiation, and their interactions on secondary metabolites, such as phenolics, terpenes, and alkaloids, continue to be studied as evidence mounting. It is important to understand those aspects of secondary metabolites that shape the success of certain plants in the future. This review aims to present and synthesize recent advances in the effects of climate change on secondary metabolism, delving from the molecular aspects to the organismal effects of an increased or decreased concentration of these compounds. A thorough analysis of the current knowledge about the effects of climate change components on plant secondary metabolites should provide us with the required information regarding plant performance under climate change conditions. Further studies should provide more insight into the understanding of multiple environmental factors effects on plant secondary metabolites.

## 1. Environmental Factors and Plant Secondary Metabolites

**Figure 1:**
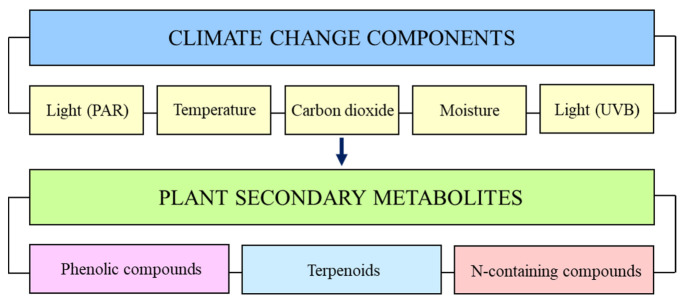
Climate change components that affect plant secondary metabolites.

Atmospheric carbon dioxide (CO_2_) concentration has reached record highs since the industrial revolution, with an annual mean concentration of 416.45 µmol mol^−1^ in 2021 [1], and an anticipated increase to 700 µmol mol^−1^ by 2100. Increases in CO_2_, along with other greenhouse gases, have led to temperature increases of 1.59 °C and 0.88 °C, over land and ocean, respectively, with a further increase of up to 5.7 °C, as projected under the very high greenhouse gas emission scenario, by the end of this century [2]. Other anthropogenic pollutants, including chlorofluorocarbons and nitrous oxide (N_2_O), have led to a reduction in stratospheric ozone, allowing a greater proportion of high-energy ultraviolet-B radiation (UVB) to reach the Earth’s surface [3] and affect other light properties, including quantity and quality. In combination, environmental stress factors are expected to strongly affect summer precipitation [2]. Together, these factors can negatively affect plant physiological processes and lead to drastic decreases in vegetative biomass and the seed yield of crop species, resulting in reduced agricultural productivity. In the current review, we report the effects of several environmental factors, including elevated CO_2_ (eCO_2_), higher temperature (HT), drought stress (DS), and ultraviolet-B radiation (UVB), on plant secondary metabolites (PSMs) (see Figure 1). As mentioned, increases in CO_2_ and other greenhouse gases in the atmosphere have led to increased global temperatures [2]. Drought and higher temperatures (HTs) are intrinsically related, where increasing temperatures cause water demands to increase, therefore leading to drought, which further increases temperature [4]. It has been shown that the components of climate change are related to each other, and their effects can be amplified throughout the rest of this century [2,4]. As projected, the current world population of 7.9 billion will reach 10.9 billion by 2100 [5]; therefore, every effort must be made to improve agricultural yield through new mitigation techniques. A good understanding of how plants will respond to climate change factors is imperative to this goal.

Through photosynthesis, plants harvest energy that leads to the production of several carbohydrate compounds, which are then used for plant metabolism and the production of primary and secondary metabolites [6]. Primary metabolites, including carbohydrates, proteins, lipids, and nucleotides, are considered essential for cell survival [6,7]. Primary metabolites lead to the production of secondary metabolites [8], which are compounds that have important roles in plants, but are non-essential to proper cellular functions and are often found to be associated with specific taxonomic groupings [7,9,10]. PSMs are important as they serve many roles in plant defense, pollinator attraction, acting as signal compounds, and abiotic stress mediation [11]. PSMs also play important roles in human society; they are used as valuable compounds in pharmaceutics, cosmetics, and nutraceuticals [9,12,13]. The concentrations of PSMs vary with both seasons and diurnal cycles, along with changes in climatic conditions [14,15]. Synthesis of PSMs requires a large carbon commitment [16], and once synthesized, they are stored in either specialized structures (trichomes) or internal organs (lactifers, resin ducts, or vacuoles) [7].

The study of plant secondary metabolism often yields variable results due to the sensitivity of metabolism. New leaves typically accumulate metabolites more rapidly than older leaves; while younger leaves have a greater nutritional content, they also have a greater defense system, both against biotic [17] and abiotic [8] stresses.

The connection between climate change components and changes in plant secondary metabolism has been of increasing interests in recent years [10,18,19]. As these factors are interconnected, a change in one component may affect other components, leading to changes in plant secondary metabolism. In the current review, we aim to highlight how individual and interactive aspects of climate change will alter plant secondary metabolism. Although the relationship between secondary metabolites and some individual environmental factors has already been reported [13], to our knowledge, the effects of multiple environmental factors have not been addressed.

## 2. Phenolic Compounds

Phenolics are chemicals that contain a phenol group, which is an aromatic ring that has a hydroxyl functional group. In this class, there are approximately 10,000 described compounds, which act in mechanical support, pathogen defense, pollinator attraction, allelopathy, and stress tolerance [8]. Phenolic compounds can also regulate growth, antioxidant activity, plant pigment, and protect leaves from incoming UV through the production of cell wall polymers [20]. Some phenolics are detrimental to livestock, as cows will avoid grass with a higher content of tannins due to their bitter taste [21], and fluctuations in these compounds can therefore have important economic implications.

Several key subclasses of phenolics include flavonoids, anthocyanins, and tannins. Flavonoids are some of the most widespread PSMs with many defensive properties, such as membrane stabilization, reactive oxygen species (ROS) quenching, enzyme inhibition, and DNA alkylation, against both abiotic and biotic stresses [22]. Anthocyanins are highly water-soluble compounds that are produced under several biotic and abiotic stresses, including UVB exposure, HTs, DS, nutrient deficiency, elevated ozone, and increased soil salinity [8]. Although anthocyanins are the final step in flavonoid biosynthesis, they may be independently regulated [23].

## 3. Terpenoids

Terpenes are the largest class of PSMs, accounting for at least 25,000 compounds [7], which are derived from the five-carbon isoprene unit. Plants lose approximately 10–20% of assimilated carbon to isoprene, even under carbon-limited environments [22]; furthermore, emissions can react with nitric oxide, contributing to the formation of tropospheric ozone [7,14]. Terpenes have a variety of important functions, such as plant communication and protection against abiotic stress, with potential roles as thermoprotectants [24,25], and some are medically important [7,24]. Notable PSMs in this category include carotenoids, carotenes, and xanthophylls, which are important both as light-harvesting pigments and antioxidants. They protect the photosystem by scavenging ROS and dissipating excess energy as heat through the xanthophyll cycle, reacting with products of lipid peroxidation, and preventing the formation of singlet oxygen [8].

Terpenes may act as membrane stabilizers, preventing proton leakage that occurs because of both an increased thylakloid membrane permeability and reduction of ATP production under higher temperatures [24,26]. Conversely, they could act as antioxidants [27], scavenging ROS species to prevent oxidative damage [26,28]. These compounds are typically stored in specialized structures, such as trichomes or resin ducts, and emissions are controlled largely by temperature and stomatal opening, as they are vaporized before emission into the atmosphere [14,24]. Stress-induced stomatal closure can cause buildup of terpenes within plant tissues [24], and since they can inhibit proper rumen microbe growth or act as analogs to neurotransmitters or hormones in humans, these compounds are unsafe for consumption [26].

## 4. Nitrogen-Containing Compounds

### 4.1. Glucosinolates

Glucosinolates are thioglucosides that contain a thiol group and a sugar that is always glucose. All glucosinolates share a common structure, with an R group that can be as simple as a single methyl group [29]. They are present in the family Brassicaceae and are economically undesirable in high quantities [7]. Glucosinolates are made from products of the shikimate pathway, including amino acids phenylalanine, tyrosine, and tryptophan, and they are formed similarly to cyanogenic glycosides [7]. Glucosinolates act as defensive compounds, which are pre-made and stored in plant vacuoles. Once released, they get cleaved by myrosinase to yield mustard oil, eventually getting broken down to form volatile defensive compounds [7,29,30]. Mustard oil has negative cellular impacts by affecting membrane fluidity and binding to target enzymes, receptors, or several other compounds [7,30].

### 4.2. Glycosides

Glycosides are compounds in which a sugar is bound to a functional group through a glycosidic bond [7]. There are several main types of glycosides, including cyanogenic glycosides, saponins, and cardiac glycosides. Cyanogenic glycosides are found in approximately 5% of all plants, acting as a constitutive defense system [31]. They are allelochemicals produced from nicotinic acid or amino acids such as phenylalanine, tyrosine, valine, or isoleucine, and are stored in plant vacuoles. These compounds are compartmentalized, but any kind of physical wounding results in decompartmentalization, beginning a chemical reaction that results in the production of hydrogen cyanide (HCN) [31]. HCN acts as a poison by blocking mitochondrial respiration and, in turn, preventing the production of ATP [30]. They are found in a wide range of plants, and occur in high concentrations in foods, such as almonds, cherries, bamboo [7], and cassava. Over-consumption of these crops can lead to cyanide poisoning, and this is already evident in parts of Africa and Asia [32].

Saponins are a class of triterpenes, some of which have sugar moieties attached through glycosidic bonds. These compounds have detergent properties and are stored in vacuoles as a form of pre-defense against pests; once released, they become hydrolyzed and can cause membrane tension and cell leakage as well as inhibit the Na^+^-K^+^—ATPase through complex formation with lipid cholesterol [30].

### 4.3. Alkaloids

Alkaloids are a group of N-containing compounds derived from pathways related to glycolysis, the tricarboxylic cycle (TCA) cycle, or the shikimate pathway, and are usually synthesized from specific amino acids [7]. There are currently 21,000 described compounds, found in approximately 20% of all vascular plants, and they share three common characteristics; all alkaloids contain at least one nitrogen atom, they are generally soluble in acidic water and organic solvents, and are biologically active in plants [7,12]. Because of their nitrogen atom, alkaloids are typically basic compounds that are stored in protonated form [7], but much of the production pathway remains poorly understood. It was once believed that these compounds were simply nitrogenous waste compounds, like urea; however, it is now known that alkaloids are important defensive compounds. Derived from the same amino acids as neurotransmitters, they can affect neuroreceptors in animals, and can also induce DNA alkylation or intercalation, cell apoptosis, or inhibit the function of key enzymes [30]. In low doses, alkaloids, such as morphine, codeine, nicotine, and caffeine, are pharmaceutically important, but in high doses, they are lethal to humans [33].

### 4.4. Non-Protein Amino Acids

Non-protein amino acids (NPAAs) are analogs of the 20 essential amino acids, present in free form. While not harmful to plants, they are detrimental to herbivores as transfer RNA is typically unable to differentiate NPAAs from actual amino acids. Thus, they are incorporated into proteins, resulting in misfolding, improper structure, and inhibition of functionality [30]. One important example of the NPAAs is γ-aminobutyric acid (GABA), which is associated with abiotic stress response along with signaling and nitrogen storage in plants. Moreover, it may act as an osmoprotectant to prevent oxidative damage during abiotic stress and plays an important role in signaling during seed and pollen germination and early imbibition in castor oil [34].

### 4.5. Polyamine

Polyamines (PAs) are N-containing small organic molecules, containing two or more amino groups, with potent biological activities in plants, including flower development, embryogenesis, organogenesis, senescence, and fruit development and maturation [35]. PAs are also important in cold acclimation responses and can lead to the biosynthesis of putrescine. Putrescine is a key molecule involved in cold stress response of Arabidopsis, leading to important compounds, such as spermidine and spermine [36].

## 5. Effects of Temperature on Plant Secondary Metabolites

As mentioned, temperature has already risen and is expected to rise more by the end of this century [2]. Earlier studies have shown that crop plants, grown under HT, had reduced yields due to decreased net photosynthesis and increased transpiration and stomatal conductance [18]. HT can lead to superficial damage of crops, including leaf and stem scorching, leaf abscission, and senescence [8]. Specific temperatures are also required for life events, such as seed germination and flowering; as such, major socioeconomic losses have occurred in the past because of extreme temperature events, and this may pose a challenge for global food security [37]. It is predicted that HT may cause a yield loss of 18–32% in potato (*Solanum tuberosum* L.), the third most important global food crop [38], and short-term heat stress irreversibly altered the biochemistry of developing anthers in barley (*Hordeum vulgare* L.) and mouse-ear cress (*Arabidopsis thaliana* L.), leading to male sterility [39].

Unlike other abiotic or biotic stresses, all cellular components of the plant sense temperature concurrently using thermosensors, which activate downstream biochemical responses [39]. While the mechanisms are not yet fully understood, it is generally accepted that heat shock transcription factor A1s (HsfA1s) are the most important factors in the heat stress response activation pathway of plants [37]. On the other end of the spectrum, plants can also experience cold stress, which includes temperatures below 20 °C, and freezing, which includes sub-zero temperatures. These temperatures can negatively affect both plant growth and distribution, resulting in agricultural losses [40]. Temperature changes induce cellular responses that lead to a number of changes in plant metabolism [11,25,38,41]. Temperature stress can also result in protein denaturation, lipid liquefaction, and the disruption of membrane integrity [11]. Down-regulation of energy and protein metabolism occur in favor of the accumulation of plant protective compounds [38]; indeed, there may be a temperature dependency of PSMs [42].

Thermal responses occur rapidly, and most temperature-related metabolite responses occur within the first 30 min of exposure [40]. Reallocation of carbon from plant growth to defense [11] could indicate maladaptive effects; while the plant would survive, it would be small and stressed, generating less yield. There is also increasing evidence that damage to cellular membranes at extreme temperature is related to a higher production of highly toxic ROS, and as such, lipid peroxidation is often used as an indicator of stress-induced oxidative damage [43]. Thermotolerance involves a synergistic effect of many compatible solutes, including hormones and several PSMs. While thermal signaling includes both cold and heat stress, these responses may have differential pathways [40]. In Arabidopsis, 497 metabolites were measured; out of these, 31% did not respond to either temperature extreme, 4% responded only to heat, 19% responded to both temperature extremes, and 38% responded only to cold [40]. Therefore, it is important to note that while one PSM may increase in response to HT, it may not necessarily decrease in response to cold stress, and these extremes will be analyzed separately.

### 5.1. Effects of Cold Stress

While poorly understood, a variety of PSMs have been shown to be influenced by cold stress. Cold stress may result in a funneling of shikimic acid into the shikimate pathway, resulting in a greater pool of metabolites. In support of this hypothesis, phenolic compounds flavonol, quercetin, kaempferol, and isorhamnetin increased with decreasing temperature in kale (*Brassica oleracea* L., var. *sabellica*) [44], flavonol content was highest in grapevine (*Vitis vinifera* L.) [23], and phenylpropanoids anthocyanins and flavonoids were also increased under cold stress in apple (*Malus* sp.) [45] (see Table 1). Increases in flavonoids may be caused by increases in sucrose, which can act as a signaling molecule to trigger flavonoid biosynthesis under lower temperatures [20].

In contrast, however, total phenolic content in Siberian ginseng (*Eleutherococcus senticosus* (Rupr. & Maxim.) Maxim.) was lower under low-temperature stress of 12 °C or 18 °C than under the ambient temperature of 24 °C [43], whereas flavanol content was significantly lower under the lower temperature treatment in pygmy smartweed (*Polygonum minus* Huds.) [25] and hypericin was lower under lower temperatures in St. John’s wort (*Hypericum perforatum* L., cv. Topas) [11], suggesting that different species may behave differently. Most terpenoids, which are responsible for the bitter taste in crops, such as carrots (*Daucus carota* L.), were found to decrease at low temperatures of 9 °C or 12 °C, except for α-terpinolene, the most abundant terpene, which increased under lower temperatures [28]. Artemisinin, a sesquiterpene, also increased under cold stress in asters (e.g., *Artemisia* sp.) [47], and these changes in terpenes may affect palatability.

In N-containing PSMs, lower temperatures enhanced quercetin glycosides but reduced kaempferol glycosides in kale (*Brassica oleracea* var. sabellica) [44], and enhanced alkyl glucosinolates glucoiberin and glucoraphanin in broccoli (*Brassica oleracea* L., var. *italica* Plenck) [42]. Cold acclimation led to an increased content of non-protein amino acid γ-aminobutyric acid (GABA) in castor bean (*Ricinus communis* L.) [34,41] and Arabidopsis [40], likely due to increases in glumatate decarboxylase, an important enzyme in the GABA biosynthetic pathway [34].

### 5.2. Effects of Heat Stress

Much like cold stress, heat stress can also result in accumulation of polyamines. In HT-sensitive cultivars of rice (*Oryza sativa* L.), putrescine levels increased under HTs, leading to a greater content of spermidine and spermine, which may scavenge radicals [50]. Temperature-sensitive cultivars may have an increased metabolic flux through the Krebs cycle due to increased concentrations of these compounds [38,50].

Regarding phenolic biosynthesis, in castor bean, an increased temperature resulted in decreased concentrations of shikimate, which increased downstream amino acid derivatives, such as tryptophan, tyrosine, and phenylalanine [41]. In Siberian ginseng (*Eleutherococcus senticosus* (Rupr. & Maxim.) Maxim.), plants that were grown under 24 °C had the highest levels of total phenolics and flavonoids; however, when the growth temperature was increased to 30°C, these levels were reduced drastically [43]. Flavonoids decreased in response to temperature in sugarcane (*Saccharum officinarum* L.) [8]. In grapevine (*Vitis vinifera* L.), HT had little effect on seed phenolics, but did reduce anthocyanins in the skin of grapes [23], likely a result of increased enzymatic degradation. In contrast, the anthocyanin content increased with temperature in sugarcane [8]. In mung bean (*Vigna radiata* (L.) R. Wilczek), it was found that plants exposed to higher temperatures had decreased flavonoids, as compared to plants exposed to lower temperatures [19]. In this case, it was suggested that the lower concentrations of flavonoids could be accounted for by the plant being unable to manage the extreme HTs, and therefore biosynthesis of flavonoids would be interrupted [19,55]. In the case of temperature-treated European aspen (*Populus tremula* L.) saplings, the concentrations of phenolic compounds, including flavonoids, phenolic acids, salicylates, salireposide, and lignan, were generally reduced, but each sex responded to the increased temperatures differently [51]. In the case of male genotypes, phenolic compound biosynthesis was found to be reduced while a dilution effect was observed in female genotypes [51] (see Table 1). Variable results were observed for other phenolics, thus phenolic compounds may have differential effects depending on pathways of biosynthesis. Since HT results in ROS accumulation, increased phenolics are adaptive as they act as ROS scavengers [8].

Temperature has an important role on terpene emissions; since volatile compounds are typically in storage form, they only reach a suitable vapor pressure under high temperature. Also, temperature is known to cause an upregulation of key enzymes in isoprene production [14]. Carrots (*Daucus carota* L.) plants that were grown at 21 °C had a higher emission of minor terpenes, and these emissions were drastically reduced at low temperatures of 9 °C, whereas major terpenes, caryophyllene and farnesene, were unaffected by temperature [28]. Similarly, sugarcane synthesized greater carotenoid levels [8] and rice grown under higher temperatures had greater emissions of terpenes [25], whereas broccoli plants that were grown under higher temperatures (>15 °C) had decreased terpenes lutein and β-carotene [42].

Increases in terpene concentrations may be an adaptive strategy by plants to increase thermotolerance; however, this advantage is limited to isoprene-synthesizing plants [24]. Under high temperature, ion permeability of the thylakoid membrane increases, which can eventually lead to a decrease in ATP synthesis due to proton leakage. It has been proposed that terpenes may help increase membrane stability to reduce proton leakage, and thus stabilize cellular structure and allow ATP production to continue [24]. Other terpenes can act as ROS scavengers at the same time, preventing lipid peroxidation in the chloroplast, and stabilizing interactions between lipids and proteins [8,26].

In shoots of St. John’s wort, napthodianthrone compounds, including hypericin, pseudohypericin, and hyperforin, were all present in the highest quantities when grown at higher temperatures [11]. GABA was also higher in Arabidopsis [40] and castor bean, like the response observed under cold temperatures. GABA, then, must react to either temperature extreme without bias; however, around 25 °C, increased catabolism of GABA into subsequent products was observed [34].

Studies are generally lacking on the effects of higher temperature on glucosinolates and glycosides; however, both year and harvest time have significant effects on glycoside content [44]. In Chinese cabbage (*Brassica campestris* L.) [44,54], higher temperatures increased kaempferol glycosides; similarly, glucosinolates content was increased in wild cabbage (*Brassica oleraceae* L.) plants that were grown under 40 °C for 15 days [49]. In broccoli, glucosinolates have been shown to increase under both cold and heat stress, suggesting that supraoptimal temperature may induce greater biosynthesis of glucosinolates [42,48,49], which can then be transported by phloem to other organs [42]. Several indole glucosinolates were unaffected by temperature, whereas glucobrassicin was found to be higher under higher temperature; since indole glucosinolates are associated with plant growth, changes in these compounds may have important agricultural implications [42]. Other N-containing PSMs, including the alkaloid ricine, increased in the cotyledons of caster bean when seedlings were grown at 35 °C, but there was a corresponding decrease in its content in the root [41].

## 6. Effects of Elevated Carbon Dioxide on Plant Secondary Metabolites

Elevated carbon dioxide (eCO_2_) affects plant growth and development traits, and often increases biomass production and yield in C_3_ crops due to carbon fertilization. CO_2_ may help mitigate the negative effects of environmental stresses on plant growth [56] but can also affect plant metabolites. Plants that are grown at eCO_2_ typically have reduced nutritional quality, nitrogen content, and protein content, potentially due to nutrient dilution [56,57]; however, response can vary with location and period of exposure [58]. Elevated CO_2_ leads to a subsequent increase in photosynthesis, producing an increased number of assimilates that are funneled into many biosynthetic pathways. This increase in photosynthesis may lead to a higher content of PSMs due to a greater availability of precursors [16]. Evidence is mounting that eCO_2_ may alter the concentrations of chemical defense substances in leaves [59], but these changes may be different for individual PSMs [60] and species.

Plant phenolics are one of the most highly studied classes of secondary metabolites in reference to eCO_2_. Out of 28 measured flavonoids, 19 increased while only three decreased and six were unaffected; similarly, out of 70 non-flavonoid phenolics, 39 increased, 11 decreased, and 20 had no effect at eCO_2_, with measurements from many different species and independent reports (see Table 2). In European aspen (*Populus tremula* L.), eCO_2_ increased total phenolics, especially salicylates and phenolic acids [51].

In flavonoids, existing reports predominantly indicate that they will increase at eCO_2_ (Table 2). Elevated CO_2_ increased total flavonoids [61,62] and tannins in most referenced studies [16,58,67,76,77,86], whereas lignins were generally unaffected [77]. Many other phenolic compounds were also increased, though a large number were unaffected; however, a study on 12 cultivars of rice showed a decrease in both total and individual phenolics [68]. Moreover, the study on rice showed that eCO_2_ decreased the overall antioxidant capacity of this species [68]. These contrasting results suggest that flavonoid content may increase in the leaves, but decrease in the fruit, which could contribute to a reduction in nutritional value.

Overall, phenolics show an increasing trend with eCO_2_; however, why this occurs is not fully understood, though CO_2_ may affect enzyme activity (Table 2). Increased phenolics could be the result of activation of transferases or hydroxylases [58], or an increase in transcription of the enzyme phenylalanine ammonia lyase (PAL), which is the first committed step in the phenylpropanoid pathway [76]. Alternatively, since increased flavonoids coincide with high sugar content, accumulation may be a result of funneling through the shikimate pathway, acting simply as a sink for an excess of photosynthates [16].

Effects of CO_2_ on terpenes seems to be less pronounced than other environmental factors [87], but there is a general trend towards increased or unaffected terpenes [76,87]. For example, lima bean (*Phaseolus lunatus* L.) [82] and cotton (*Gossypium hirsutum* L.) [78,79] had increased terpene concentrations, whereas terpenes were inhibited in oak (*Quercus Ilex* L.) [27], which could indicate differential responses between herbaceous and woody species. CO_2_ had no effect on carotenoid content in tomato (*Solanum lycopersicum* L.) [81] and sesquiterpenes were unaffected in tobacco (*Nicotiana tobaccum* L.) [76].

N-containing PSMs are often toxic and act in smaller quantities than C-based PSMs. The effect of CO_2_ on N-containing PSMs has received little attention; however, it has been hypothesized that these compounds would be reduced because of decreased overall N-content in leaves [32,88]. Independent research papers do not agree with this hypothesis, as several studies have shown that N-containing PSMs have increased under elevated CO_2_. N-containing PSMs could decrease the palatability of important crops and increase the toxicity to herbivores, including humans [89]. Effects of CO_2_ on glucosinolates were marginal and inconsistent among cultivars in canola (*Brassica napus* L.). However, total glucosinolates were decreased with decreases in individual indole glucosinolates, such as glucosbrassicin [29]. Observed decreases could be due to a dilution effect with higher biomass as opposed to a decreased biosynthesis. While this reduction would benefit palatability, it would have a negative effect on constitutive plant defense [29].

Cyanogenic glycosides were increased in two cultivars of red lettuce (*Lactuca sativa* L. var. crispa L. cv. Eventai RZ and *Lactuca sativa* L. var. crispa L. cv. Satine) [16] and in the leaves of cassava (*Manihot esculenta* Cranz.), but not in its tubers, when grown under elevated CO_2_. In cassava, cyanogeic glycoside increased from 0.7% of total leaf N to 1.5%, which may be responsible for reallocation of leaf N away from photosynthesis [32]. While cyanogenic glycoside concentration did not significantly increase in white clover (*Trifolium repens* L.), there was a 40% decrease in total protein content, which increased the proportion along with overall toxicity [31]. Similar effects were observed in young ginger [67], which had an increased concentration of cyanide.

In alkaloids, compounds such as indole alkaloids, nicotine, and 5-O-caffeoyl-D-quinic acid (CGA) were increased with increased CO_2_ [59,76], possibly because of decreased production of primary metabolites [76]. Generally, elevated CO_2_ resulted in increased alkaloids, which could positively influence commercial-scale production of these metabolites. Under elevated CO_2_, significantly more morphine, codeine, papaverine, and noscapine were produced in wild poppy (*Papaver setigerum* L.) [33], and higher concentrations of scopolamine were accumulated in jimson weed (*Datura stramonium* L.) [84].

## 7. Effects of Drought Stress on Plant Secondary Metabolites

Drought stress (DS) can result from decreased available water, either in the short or long term, resulting from low-precipitation or increased evaporation [90], and often leads to reduced plant growth and yield [91]. Irrigation and maximization of crop efficiency is a global issue, since at least 70% of available water is used in agriculture and more than 40% of global agriculture uses irrigated soils. Changes in water availability can therefore cause severe economic losses [92]. Plants are not always severely harmed by DS; instead, acclimation can happen slowly over a few weeks, leading to metabolic adjustments and reduced overall growth [90,93].

Like any other stress condition, there is a species-specific set of responses to DS, with at least 30 different metabolites currently known to respond to a water deficit [94]. Since key components of the plant antioxidant machinery may be reduced because of severe stress, DS-induced plant responses are critical for survival [95], especially in higher plants, such as crops, where drought tolerance is uncommon [92]. The first response of plants to DS is the imbalance of osmotic potential [93], and almost all plants can acclimate to moderate DS through maintenance of cell turgor pressure [95]. The other primary response is the accumulation of solutes, including PSMs, because of a lower influx of CO_2_, which decreases consumption of Calvin cycle reduction equivalents [96]. These compounds, which include proline and polyols, surround the hydration shells of sensitive proteins, thus preventing osmotic stress-induced protein degradation [94].

In about half of the reported cases, flavonoids increased in response to drought, whereas about two-thirds of non-flavonoid phenolic compounds increased under DS (see Table 3). Phenolic compounds may only increase in drought-tolerant cultivars, with decreases in sensitive cultivars. This was observed in maize (*Zea mays* L.), where total phenolic content increased only in tolerant genotypes, but not in sensitive or intermediate ones [97]. Individual or total phenolics were also increased in other drought-tolerant plants, such as cumin (*Cuminum cyminum* L.) [98], peanut (*Arachis hypogaea* L.) [99], and “milfacadas” (*Hypericum Brasiliense* Choisy), a plant native to southern Brazil that is accustomed to water-limited environments [46]. Even on a per-plant basis, DS plants were smaller, but had a 10% increase in per-plant phenolic content in comparison to the control [46]. By contrast, phenolics decreased in cotton (*Gossypium* sp.) [100], tea (*Camellia sinensis* L.) [101], and seeds of developing oat (*Avena fatua* L.) [102]; these indicate that these cultivars are drought sensitive.

Like phenolics, terpenoids exhibit high variability in response to drought, with only 35 of 50 terpenes increased in response to DS (Table 3). In thyme, terpene increases are transient, with peak increases around 2 weeks following onset of DS, which eventually levels off and may decrease in the long-term [92]. Therefore, differential growth periods and testing times may account for some of the variability among studies. Many studies examining terpenes under these conditions used woody species, such as trees, which may not be representative of agricultural crops. In liquorice (*Glycyrrhiza glabra* L.), glycyrrhizin and the genes that enhance the production of PSMs, such as triterpene saponins, increased under DS [117]. Monoterpenes were increased in thyme in response to DS [92]. Sesquiterpenes were increased in sweet wormwood (*Artemisia annua* L.), while other sesquiterpenes decreased in the same species, proportional to the severity of DS [114]. A potential reason for decreased terpene emissions in some species is the effect of DS on glandular trichomes; these structures act as storage for several terpenes, including artemisinin. Trichomes were shown to decrease in length with increasing DS [114] and decreases in emissions may be directly proportional to the loss in storage of glandular trichomes. Carotenoids were also shown to decrease in many cases [112,114,115], except in nasturtium (*Tropaeolum majus* L.) [96].

Since antioxidant defense is impaired under DS, plants can increase xanthophyll pigments to eliminate excess energy through thermal dissipation. Increases in xanthophyll pigments can preserve thylakoid membranes by avoiding ROS generation; however, under severe drought, increases in zeaxanthin are insufficient to preserve the photochemistry of PSII, and eventually drought-induced impairments can depress enzymatic activity and lead to photoinhibition [112].

Soil water content is a key factor regulating N-containing PSMs, and since glucosinolates are responsible for the recognizable taste of crops, including mustard, horseradish (*Armoracia rusticana* Gaertn., C.A. Mey. & Scherb.), and nasturtium (*Tropaeolum majus* L.) [96], drought stress can have significant implications on the growth of these crops. Drought stress of 20–37% of field capacity led to a water content on the brink of the permanent wilting point in nasturtium [96], and at this level, glucosinolates have been shown to increase in the seeds of canola [118] and leaves of Ethiopian kale (*Brassica carinata* (A.) Braun) [119] (Table 3). Drought stress also increased the content of cyanogenic glycosides on a per-plant basis in lima bean (*Phaseolus lunatus* L.) [82].

In most plants, alkaloids have been shown to increase [123], which is consistent with the examined studies, as 13 out of 14 described alkaloids increased with DS (Table 3). Accumulation of alkaloids was increased in celandine (*Chelidonium majus* L.) [92], tobacco [124], potato [121], Madagascar periwinkle (*Catharanthus roseus* L.) [123], soybean [122], and ragwort (*Senecio jacobeae* L.) [125]. In celandine, increases were restricted to benzyl isoquinoline alkaloids, but these increases were transient and subsequently decreased over time [93].

Along the same lines, GABA was shown to increase in several herbaceous and woody plant species [90,95,126,128] but was shown to be unaffected in wheat [93]. However, the polyamine putrescine decreased under long-term DS in rice [126].

## 8. Effects of Light on Plant Secondary Metabolites

Light is one of the most important environmental factors that determine plant growth and success [129,130], and this includes several factors, such as light intensity, duration, and quality. All plants have photoreceptors that can detect aspects of light through a process called photomorphogenesis [131]. From incoming light, plants absorb approximately 90% of red and blue light, making them sensitive to any alteration in light environment [132]. Both light quantity and quality can influence plant morphology, development, and the synthesis of both primary and secondary metabolites [133], often acting in a species-specific manner [129]. Also, light is a critical component of cellular differentiation, including expansion of hypocotyls, chloroplast development, leaf expansion, and initiation of flowering [134]. Blue light can stimulate phototropism, shortening of stems, movement of chloroplasts, regulation of stomata, and genetic expression [131].

It is widely understood that light intensity has the potential to positively affect phytochemical accumulation, but light quality leads to more complex responses that can differ among species and light ratios [133]. Light activates many important metabolites, such as phenolic compounds, which can act as antimicrobial or antifungal agents along with ROS scavengers [135]. Also, carotenoids, important tetraterpenoid pigments, are in close association with the thylakoid membranes of chloroplasts; through interactions with photosystem (PS) complexes I and II, carotenoids assist in energy capture along with dissipation of extra energy as heat [131].

Light-induced PSMs can either act as direct defense compounds, or can be present in pre-defensive compounds, ready to be used in biosynthetic processes. In Arabidopsis, monolignol glucosides were shown to accumulate under increased light, acting as mobile precursors of coniferyl and syringyl alcohol, both of which are critical in the biosynthesis of lignin [134]. Also, light stress can activate the accumulation of certain PSMs in relevant areas; in manna ash (*Fraxinus ornus* L.), antioxidant coumarins were found to accumulate in the outer edges of vacuoles in sun-adapted leaves, whereas poor antioxidants, such as esculetin, were found deeper within the vacuoles [136]. Light, therefore, has an important role in plant metabolism, leading to an increased C:N balance and increased PSM production, even under reduced photosynthetic capacity [113].

### 8.1. Light Quantity

Sunlight is a big driver of phenolic biosynthesis, and this may act on a diurnal clock, increasing the importance of photoperiod in addition to light intensity. Increases in individual phenolics were observed in Arabidopsis [134] and potato [137], whereas a decrease in total phenolics was found in Arabidopsis [134], cat’s whiskers (*Orthosiphon stamaneus* Benth.) [113], and shade-intolerant medicinal plant jewel orchid (*Anoectochilus formosanus* Hayata); therefore, increased light may have negative consequences on PSM production in sensitive plants [132]. Longer photoperiod increased phenolics in sweet basil (*Ocimum basilicum* L.) [138] and cyanogenic glucosides in buckwheat (*Fagopyrum tataricum* L.) [139]; conversely, most phenolics decreased under low light in some species, including rice [140], American tarwort (*Flourensia cernua* DC.) [129] and sweet basil [138].

A general trend toward increased phenolic production under high light and decreased phenolics under low light (see Table 4) may be regulated on a genetic level, through increased transcription of both the phenylpropanoid pathway [134] and of other important defensive pathways. Not all phenolics are similarly regulated; in Arabidopsis, opposite changes are observed in soluble and insoluble phenylpropanoids, which may be the result of upregulation of certain portions of the phenylpropanoid pathway, but this process is poorly understood [134].

While relatively understudied, both terpenoids and N-containing PSMs appear to increase under high light and low light (Table 4). Isoprene emissions are typically stimulated during transient events, such as drought or heat waves [22], and this may apply to light changes as well. Carotenoids were increased under higher light in field mustard (*Brassica rapa* L.) but decreased in Indian mustard (*Brassica juncea* L. Czern) [150]. Since carotenoids protect photosynthetic machinery from excess light, it is expected that these compounds should increase with light in tolerant species [22].

In N-containing PSMs, aliphatic glucosinolates were higher under a longer photoperiod in wild cabbage (*Brassica oleracea* L.), whereas the indole glucosinolates were unaffected [49]. White mustard (*Sinapis alba* L.) also showed increased glucosinolates with increasing photoperiod up to 22 h [151]. Similarly, longer photoperiods increased the content of GABA [139], glycosides, and alkaloids, including the glycoside ginsenosides in American ginseng (*Panax quinquefolius* L.) [152] and the alkaloid solanine in potato [153]. When grown under shade, some plants had higher cyanogenic glycosides, whereas others had decreased cyanogenic glycosides. Variability was also observed in alkaloids, which increased under shade in evergreen tropical tree (*Tabernaemontana pachysiphon* Stapf) [146] but decreased in the herb subalpine larkspur [147]. Conversely, light had no effect on potato plants in two independent studies [144,154]. Overall, higher light intensity appears to increase N-containing PSMs, whereas lower light levels decrease them (Table 4). Since N-containing compounds require a higher energy investment [135], favoring C-based defensive compounds, such as phenolics, under shade conditions may be an adaptive mechanism.

### 8.2. Light Quality

Effects of light quality on PSMs are highly variable, depending not only on species, but also on the specific ratios between blue (B), red (R), and far-red (FR) lights, along with whether lights are incandescent or fluorescent [157]. Many studies have simply focused on blue light. Blue light significantly increased flavonoid content in all 12 examined cases, whereas other phenolic compounds were increased in 12 of 17 cases (Table 4); this up-regulation may help as a pre-conditioning to help the plant in the event of stressful environmental conditions [135]. Increases in phenolics, including flavonoids, may be promoted by an increase in enzymatic activity. When grown under red light, rosmarinic acid, a phenolic compound, was increased in basil (*Ocimun basilicum* L.) [138] and p-coumaric acid was the only phenolic that increased in ginseng [157]. Yellow and green lights also increased phenolics and flavonoids in selfheal (*Prunella vulgaris* L.) [130].

Terpenes were increased by blue light in most observed cases (Table 4). Red light stimulated biosynthesis of curcubitacin, an important terpene in agarwood (*Aquilaria malaccensis* L.), but it was decreased with a simultaneous increase in FR content. Similarly, FR light also decreased xanthophylls and carotenes in lettuce [53]. Green light increased concentrations of terpenes perillaldehyde and limonene, and this was likely due to a decrease in overall plant biomass as the per-plant terpene concentration was lower under green light than under blue and B:R treatments [156].

All N-containing compounds, including six glucosinolates and three glycosides, were increased by blue light. As little as 5 days of blue-light application was enough to increase both aliphatic and aromatic glucosinolates in broccoli [131]. Both blue and red lights also induced accumulation of the glycoside eleutheroside B in Siberian ginseng [43]. Red light also induced accumulation of the glucosinolate gluconasturtiin in watercress (*Nasturium officinale* W.T. Aiton) [53]. In some studies, plants grown under blue lights did not have a higher biomass, but instead assimilates partitioned differently because of enzymatic responses differing between species and cultivars [156].

## 9. Effects of Ultraviolet-B Radiation on Plant Secondary Metabolites

Chlorofluorocarbons and nitrous oxide (N_2_O) have caused a reduction in stratospheric ozone [3], leading to an increase in UVB radiation (280–320 nm) reaching the Earth’s surface [21,162]. Based on the current estimates, increases in surface-level UVB have been occurring at approximately 2–5% per decade in Europe; recovery of the ozone layer is expected by the mid-21st century [21]; however, UVB is also affected by factors such as cloudiness, altitude, and latitude. Though only a small portion of the spectrum, UVB can cause a variety of photobiological effects [162], and above-ambient levels can lead to the accumulation of free radicals and ROS. UVB is sensed by a specialized receptor called the UV Resistance Locus 8 (UVR8) cellular component [163]; when presented in realistic quantities, UVB may not be stressful to plants, as it is required for processes, such as gene expression, metabolic activities, and plant growth and development [164,165]. Like those of other environmental factors, the effect of UVB on plants depends, in part, on interactions with other factors; for example, sensitivity of white clover depends on water availability and plant genetics [166]. UVB is one of the most well-studied of environmental factors on plant metabolism (see Table 5).

PSMs can serve as primary antioxidants at the initiation of UVB stress, and it is generally known that C-based metabolites, including phenolics and especially flavonoids, are stimulated by UVB radiation [129,162]. This was found in most cases (Table 5), even when radiation was only applied for a few days [172]. Flavonoids are critical to UV tolerance as they absorb harmful rays, preventing them from being further absorbed into the plant cells [172]. Arabidopsis mutants lacking the flavonoid production mechanisms are hypersensitive to UVB radiation, whereas those with amplified flavonoid production are tolerant to typically lethal UVB levels [173]; however, different classes or structures may respond differently. Two of the main categories of flavonoids include quercetin glycosides and kaempferol glycosides; biosynthesis of quercetin glycosides may be favored under supplemental UVB [162,178], regardless of kaempferol being more effective at UVB absorption, because quercetin is a more efficient ROS scavenger [178]. By contrast, UVA stimulates a greater production of kaempferol derivatives to favor absorption of harmful rays [155]; therefore, if the UV ratio changes, phenolic biosynthesis will be affected as well.

In tomato, monosubstituted flavonols were increased under UVB whereas trisubstituted flavonols were unaffected [181]. Supplemental UVB also increased tannins in some species [170] but decreased it in others [187]. Lignin was shown to increase in most species, including grapevine [185], coleus (*Coleus forskohlii* L.) [183], Indian ginseng (*Withania somnifera* L.) [182], and linseed (*Linum usitatissimum* L.) [186]. Several other flavonoids were unaffected [155], but this represents a small minority (Table 5). Since protein content is reduced under UVB stress, phenol enhancement could be representative of a trade-off between polypeptide and phenol biosynthesis [183]. Increases in phenolics can also be the direct result of ultraviolet activation of specific genes, including those involved in committing steps of the phenylpropanoid pathway [162,164,165].

While volatile organic carbons do not appear to change in response to UVB, monoterpenes typically increase at either end of the spectrum [129,187], and this may depend, in part, on developmental stage. Carotenoids are unaffected in long-term studies [21], but acute UVB exposure typically leads to increased carotenoid content through genetic upregulation, while chronic exposure leads to genetic down-regulation [165]. On the same note, some xanthophyllic pigments are unaffected by chronic UVB [167], while others, such as violaxanthin, neoxanthin and zeaxanthin, are decreased due to a reduction in abscisic acid synthesis [188].

N-containing compounds of all sub-classes, except glucosinolates, increase in response to UVB. Glucosinolates, however, only increased in 11 of 27 cases (see Table 5). These results can be variable because glucosinolate content differs between plant organs, age, and developmental stage [17,119], and UVB dose and duration [191]. Alternatively, these responses may be transient since studies have shown that initial increases in glucosinolates were short-lived and resumed normal levels soon after [191]. Conversely, glycosides are rapidly accumulated upon UVB exposure, and this response lasts longer than that of glucosinolates. UVB exposure increased cyanide production in white clover due to increased production of cyanogenic glycosides [88], and increased pools of glycosides were also observed in Arabidopsis [178]. Since glycosides can be readily metabolized to flavonols and relocated to relevant sub-cellular compartments, this is an efficient metabolic storage system [178]. Other studies, however, have proposed that long-term effects of UVB may level out glycosides similarly to glucosinolates [21].

A study with the sugar beet (*Beta vulgaris* L.) plants, using three doses of UVB radiation (3, 6 and 9 kJ m^−2^ d^−1^), has shown that plants exposed to the highest dose had significantly decreased growth and biomass, but increased stress-related chemicals, including total betalain [198].

A total of 27 alkaloid compounds were found that were measured under supplemental UVB radiation, with many of these compounds increasing (Table 5), including catharanthine in periwinkle (*Catharanthus roseus* L.) [196], withaferin A in Indian ginseng [182], and brachycerine in a native shrub of Brazil (*Psychotria brachyceras* Müll. Arg.) [194]. Supplemental UVB can also induce the accumulation of new alkaloids, as three new alkaloids were found in cauliflower (*Brassica oleracea* L.) under UVB treatment [184]. Induced alkaloid accumulation may not be beneficial; higher content of nicotinamide and other alkaloids in tobacco can lead to cellular damage, while other compounds, such as the indole alkaloids, accumulate due to antioxidative properties [184].

Polyamines are less understood regarding their response to supplemental UVB [21]. Increases in polyamines were found following acute exposure to UVB, but these responses decreased as time passed [91,195]. Transient increases were also found in thylakoid-associated polyamines, which may act as a temporary solution [21], accumulating where ROS are produced as a first line of defense [178]. This primary defense may be helped by non-protein amino acid GABA, which also accumulates in response to UVB [181].

## 10. Interactive Effects of Environmental Factors on Plant Secondary Metabolites

Many studies have examined the relationship between individual stress factors and specific classes of PSMs; however, while this information provides valuable insight about each stress factor, abiotic stress conditions under natural conditions are much more complex. At any given time, various stress factors may occur together; HL often comes with increased temperatures, eCO_2_ with HT, HT with DS, and HL typically involves both visual light and ultraviolet light [90]. These studies, however, are generally lacking, and therefore, information regarding the interactive effects of stress factors on PSMs is scarce.

A recent study on barley (*Hordeum vulgare* L.) has shown that some PSMs may be negatively affected by a combination of HT and eCO_2_ [199]. In silver birch (*Betula pendula* Roth), the concentration of chlorogenic acid was unaffected by eCO_2_ alone; however, a 2–3°C increase resulted in a significantly decreased concentration, and a similar trend was observed for both chlorogenic acid and feruloyl quinic acid [86] (see Table 6). In cotton (*Gossypium hirsutum* L.), HT with eCO_2_ increased total leaf phenolics, but with ambient CO_2_ (aCO_2_), it decreased phenolic concentrations [80]. However, in European aspen (*Populus tremula* L.), HT with eCO_2_ decreased phenolics [51]. In tall fescue (*Schedonorus arundinaceus* (Schreb.) Dumort.), HT with eCO_2_ resulted in decreased GABA content, whereas GABA was increased under HT with aCO_2_ [200]. When temperature occurred in combination with HL, biosynthesis of anthocyanins was down-regulated in grape berries through reduction of phenylalanine ammonia-lyase (PAL) enzyme activity; however, moderate temperature was shown to enhance anthocyanins and alter the ratio between anthocyanins and flavonol-glycosides, which are important for ROS-scavenging abilities. By contrast, GABA was accumulated by an increased flow through the GABA shunt [201]. A study on kacip fatimah (*Labisia pumilia* Benth.) suggested that increased carbohydrate content may get channeled for increased PSM production [142]. HT and DS also induced biosynthesis of flavonoids, catechins, and sitosterol content in Douglas fir (*Pseudotsuga menziesii* (Mirb.) Franco) [202]. In mouse-ear cress (*Arabidopsis thaliana* L.), drought-stressed plants that were grown under HT at aCO_2_ produced seeds with decreased total phenolics, whereas drought-stressed plants that were grown under lower temperature (LT) at eCO_2_ produced seeds with increased phenolics [52] (see Table 6).

On the other hand, glucosinolate responses to HT and HL vary with type. HT with longer days reduce glucoiberin, whereas lower temperatures (LT) with the same photoperiod induce accumulation of aliphatic and aromatic glucosinolates, including glucoraphanin, sinigrin, and gluconasturtiin [209]. This may be a result of seasonal variation in PSMs among species, such as turnip, radish, canola, and mustard, with most of these showing the greatest glucosinolates content in the spring, with moderate temperature, HL, longer days, and dryer conditions, in comparison to autumn’s lower temperature, HL, shorter days, and higher water availability, which lead to the lowest content [210]. Isoprene emissions are also dependent on temperature and light. Isoprene emitted under sunlight increases exponentially with temperature, up to a maximum of approximately 40 °C, and following this, dysregulation of the system may occur [26].

Under HT and DS, photosynthesis declines because of stomatal limitations, and these conditions may favor isoprene production and emissions [22]. Emission of isoprene increased until 45 °C in date palm (*Phoenix dactylifera* L.), a tolerant plant, but when plants were under DS, isoprene increased only to a temperature of 40 °C, following which it decreased dramatically. Below 40 °C, isoprene contributes to thylakoid membrane stability and increased fatty acid content, which indicates that severe heat stress may have negative consequences [207]. HT and DS also induced biosynthesis of flavonoids, catechins, and sitosterol content in Douglas fir (*Pseudotsuga menziesii* (Mirb.) Franco) [202].

PSMs accumulated in Kacip Fatimah (*Labisia pumila* Benth.) when plants were grown under eCO_2_ and low light (LL), and this was a consequence of increased enzymatic activity. Total phenolics and flavonoids were similarly increased under these conditions, along with anthocyanins. In contrast, eCO_2_ and HL reduced PAL activity, which suggests that eCO_2_ may counteract the effects of HL on phenylpropanoid enzymes [142]. When eCO_2_ is combined with UVB, enzyme activity is also increased, including that of PAL, leading to accumulation of condensed tannins, total phenolics, and cinnamic acid derivatives in birch (*Betula pendula* Roth). A combination of these factors increased resource allocation per leaf on a dry mass basis, but the results varied among individual PSMs [204].

In the absence of impact from temperature, CO_2_, or DS, UVB and blue light applied simultaneously to lettuce plants stimulated the phenylpropanoid pathway, resulting in a greater downstream accumulation of metabolites [206]; however, many individual compounds respond differently [211]. HL and UVB induced quercetin accumulation in Brazilian pennywort (*Hydrocyte leucocephela* Cham. & Schltdl.) [205] and Asiatic pennywort (*Centella asiatica* L.) [211], but HL decreased the total kaempferol concentration, leading to a higher quercetin/kaempferol ratio, as observed in plants grown under UVB alone [205]. Similarly, all saponins accumulated under HL, although this was not necessarily dependent on ambient or supplemental UVB levels. Flavonols and anthocyanins were increased under HL and UVB, along with accumulation of the carotenoid zeaxanthin under HL and DS, both of which protect leaves, either through prevention of harmful light penetration or irreversible chloroplast damage [205].

## 11. Future Perspectives and Concluding Remarks

Climate change components induce metabolic changes in plants, altering both primary and secondary metabolism. However, studies that examine multiple stress factors on plants are scarce, despite plant responses to them in natural habitats, and their differential effects than those of individual factors [18]. For instance, eCO_2_ may mitigate the effects of DS [115], whereas a combination of HT and DS is more detrimental than either stress alone [207]. As shown, barley (*Hordeum vulgare* L.) plants that were grown under single stress factors could adapt, becoming more resistant, whereas a combination of climatic factors neutralized this effect [199]. Similarly, tomato (*Solanum lycopersicum* L.) plants perform better when grown under a combination of higher temperature and light as opposed to either factor alone [212]. In nature, the plant stress response is a well-orchestrated signaling event as the result of thousands of years of evolution. Plant tolerance to abiotic stress is not mediated by a single factor.

**Figure 2:**
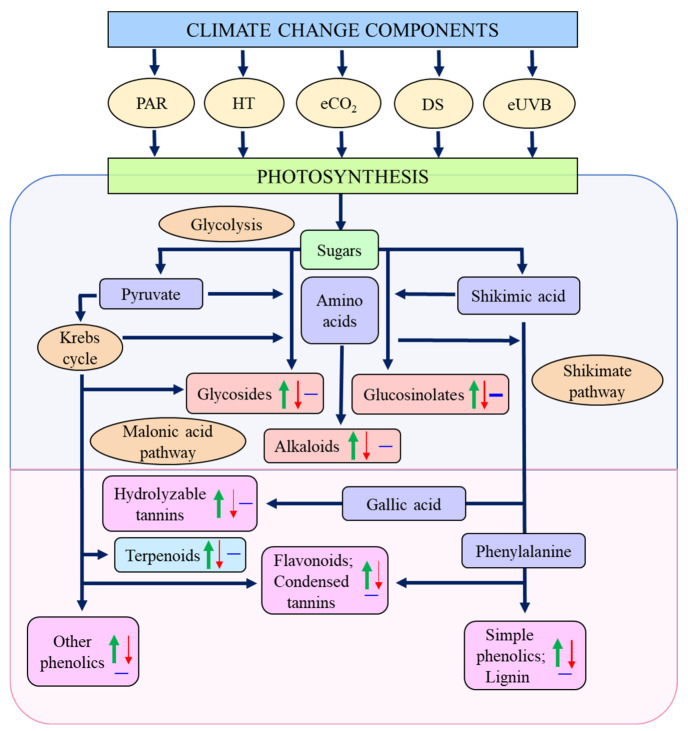
Biosynthesis of plant secondary compounds from primary metabolites, as affected by climate change components. Upward arrows, increase; downward arrows, decrease; horizontal lines, no effect; PAR, photosynthetically active radiation; HT, higher temperature; eCO_2_, elevated carbon dioxide; DS, drought stress; eUVB, enhanced ultraviolet-B radiation. Data are derived from the literature cited in Table 1, Table 2, Table 3, Table 4, Table 5 and Table 6, and the metabolic pathways are based on references [6,12].

Analysis of the current studies on the interactive effects of abiotic factors on PSMs has shown a general increase of these chemicals (Table 6; Figure 2). In secondary metabolism, it is noteworthy that the only case in which PSMs decreased was under eCO_2_; since CO_2_ reduces protein and nutritional content of crops [56], it may lead to the inhibition of important biosynthetic pathways, through limitations in precursor metabolites or reduced enzymatic capacity for key enzymes, such as PAL [76]. On the other hand, eCO_2_ increased toxic components in other plants [67], suggesting that negative impacts on humans may be exacerbated, but it remains unknown whether CO_2_ will have the same effect when in co-occurrence with other abiotic stresses.

Increases in the production of PSMs is important for plant survival, as these compounds have defensive roles against a broad range of environmental stresses. Increases in PSMs can be considered positive or negative based on agricultural aspects. Some secondary metabolites, such as phenolic compounds and flavonoids, are antioxidants and important in the human diet. PSMs are also used for the treatment of ovarian and breast cancers, heart disease (e.g., terpenoids), arthritis and other inflammatory diseases (e.g., phenolics), as well as for the dilation of the pupil during eye examination and antimalarial treatment (e.g., alkaloids). Others, however, can have a variety of toxic effects, including protein denaturation, DNA alkylation, and in some extreme cases, chronic cyanide poisoning [9,12,13]. It becomes important, then, to understand how these metabolites will respond to multiple realistic stress factors, to assess the impacts that climate change components will have on plant metabolism. Understanding these interactions will provide insight into one of the fundamental issues facing the global population, the ability to increase nutritional content and agricultural yield while minimizing undesirable compounds.

## Figures and Tables

**Table 1:** Effects of temperature on plant secondary metabolites.

Class		Sub-Class	Metabolite Number	Effect	References
Cold stress					
	Phenolics	Flavonoids	13	11(I); 1(D); 1(NE)	[20,23,25,44,45]
		Others	3	2(I); 1(D)	[40,44,46]
	Terpenoids	-	10	2(I); 7(D); 1(NE)	[25,28,47]
	N-containing compounds	Glucosinolates	2	2(I)	[48,49]
		Glycosides	2	2(I)	[44]
		Alkaloids	5	5(I)	[36,40]
		Others	1	1(I)	[40]
Heat stress					
	Phenolics	Flavonoids	5	3(I); 1(D); 1(NE)	[8,23,25,43]
		Others	16	5(I); 10(D); 1(NE)	[8,11,19,38,40,43,46,50,51,52]
	Terpenoids	-	27	18(I); 8(D); 1(NE)	[8,25,28,42,46]
	N-containing compounds	Glucosinolates	11	4(I); 2(D); 5(NE)	[42,48,49,53]
		Glycosides	1	1(D)	[54]
		Alkaloids	4	2(I); 2(D)	[34,40,41,50]
		Others	5	4(D); 1(I)	[11,34,40,41]

Data are derived from the references shown. (I), increased; (D), decreased; (NE), no effect.

**Table 2:** Effects of elevated CO_2_ on plant secondary metabolites.

Class	Sub-Class	Metabolite Number	Effect	References
Phenolics	Flavonoids	28	19(I); 3(D); 6(NE)	[16,59,61,62,63,64,65,66,67,68,69]
	Others	70	39(I); 11(D); 20(NE)	[16,51,52,58,59,60,67,68,69,70,71,72,73,74,75,76,77,78,79,80]
Terpenoids	-	23	9(I); 4(D); 10(NE)	[27,63,67,72,75,78,79,81,82]
N-containing compounds	Glucosinolates	17	6(I); 7(D); 3(NE)	[29,69,83]
	Glycosides	7	3(I); 2(D); 2(NE)	[16,31,32,67,73,74]
	Alkaloids	12	8(I); 4(D)	[33,59,76,84]
	Others	2	1(I); 1(D)	[85]

Data are derived from the references shown. (I), increased; (D), decreased; (NE), no effect.

**Table 3:** Effects of drought stress on plant secondary metabolites.

Class	Sub-Class	Metabolite Number	Effect	References
Phenolics	Flavonoids	21	10(I); 5(D); 6(NE)	[46,91,92,95,103,104,105,106,107]
	Others	38	28(I); 9(D); 1(NE)	[46,91,97,98,99,100,101,102,103,105,106,107,108,109,110,111,112,113,114]
Terpenoids	-	50	35(I); 11(D); 4(NE)	[92,96,99,103,104,111,112,114,115,116,117]
N-containing compounds	Glucosinolates	3	3(I)	[96,118,119]
	Glycosides	4	3(I); 1(NE)	[82,103,107,120]
	Alkaloids	14	13(I)	[90,92,121,122,123,124,125]
	Others	4	3(I); 1(D); 1(NE)	[93,126,127,128]

Data are derived from the references shown. (I), increased; (D), decreased; (NE), no effect.

**Table 4:** Effects of light quantity and quality on plant secondary metabolites.

Class		Sub-Class	Metabolite Number	Effect	References
Low light					
	Phenolics	Flavonoids	10	2(I); 8(D)	[113,139,140,141,142]
		Others	9	4(I); 5(D)	[83,113,129,138,143,144]
	Terpenoids	-	12	8(I); 4(NE)	[129,141,145]
	N-containing compounds	Glycosides	3	3(D)	[143]
		Alkaloids	5	1(I); 3(D); 1(NE)	[144,146,147]
		Others	1	1(D)	[139]
High light					
	Phenolics	Flavonoids	4	2(I); 2(D)	[43,113]
		Others	8	5(I); 2(D); 1(NE)	[43,113,134,137,148]
	Terpenoids	-	4	3(I); 1(D)	[8,149,150]
	N-containing compounds	Glucosinolates	3	2(I); 1(NE)	[49,151]
		Glycosides	1	1(I)	[152]
		Alkaloids	5	3(I); 2(NE)	[137,144,153,154]
Blue light					
	Phenolics	Flavonoids	12	12(I)	[130,135,155,156]
		Others	17	12(I); 2(D); 3(NE)	[130,133,134,135,138,156,157]
	Terpenoids	-	13	12(I); 1(D)	[155,156,157,158,159,160]
	N-containing compounds	Glucosinolates	6	6(I)	[159,161]
		Glycosides	3	3(I)	[43,135,140]

Data are derived from the references shown. (I), increased; (D), decreased; (NE), no effect.

**Table 5:** Effects of ultraviolet-B radiation on plant secondary metabolites.

Class		Sub-Class	Metabolite Number	Effect	References
Enhanced					
	Phenolics	Flavonoids	80	45(I); 3(D); 17(NE)	[17,155,158,161,162,163,166,167,168,169,170,171,172,173,174,175,176,177,178,179,180,181,182,183]
		Others	61	40(I); 16(D); 5(NE)	[17,119,127,158,162,163,164,167,169,170,171,174,175,176,178,179,182,183,184,185,186]
	Terpenoids	-	36	24(I); 8(D); 3(NE)	[21,158,163,167,176,179,182,183,187,188,189,190]
	N-containing compounds	Glucosinolate	27	11(I); 4(D); 12(NE)	[17,21,119,161,191]
		Glycosides	13	10(I);1(D); 2(NE)	[17,21,88,155,177,182,183]
		Alkaloids	27	20(I); 7(NE)	[155,178,182,183,184,192,193,194,195,196]
		Others	5	5(I)	[181,197,198]

Data are derived from the references shown. (I), increased; (D), decreased; (NE), no effect.

**Table 6:** Effects of multiple environmental factors on plant secondary metabolites.

Factor	Species	Compound	Effect	Reference
Phenolics				
HT × eCO_2_	*Betula pendula* Roth	Chlorogenic acid	Decreased	[86,203]
HT × eCO_2_	*Gossypium hirsutum* L.	Total phenolics	Increased	[80]
HT × eCO_2_	*Vitis vinifera* L.	Anthocyanins	Decreased	[201]
HT × DS	*Pseudotsuga menziesii* (Mirb.) Franco	Flavonoids	Increased	[202]
eCO_2_ × LL	*Labisia pumila* Benth.	Flavonoids, anthocyanins, total phenolics	Increased	[142]
eCO_2_ × UVB	*Betula pendula* Roth	Tannins, total phenolics	Increased	[204]
HL × UVB	*Hydrocotyle leucocephela* Cham. & Schltdl.	Quercetin	Increased	[205]
HL × UVB	*Hydrocotyle leucocephela* Cham. & Schltdl.	Flavonols, anthocyanins	Increased	[205]
UVB × BL	*Lactuca sativa* L.	Phenylpropanoids	Increased	[206]
HT × aCO_2_ × DS	*Arabidopsis thaliana* L.	Total phenolics	Decreased	[52]
LT × eCO_2_ × DS	*Arabidopsis thaliana* L.	Total phenolics	Increased	[52]
Terpenoids				
HT × DS	*Phoenix dactylifera* L.	Isoprene	Increased	[207]
HT × HL	*Phragmites australis* (Cav.) Trin. ex Steud.	Isoprene	Increased	[26]
HL × DS	*Xerophyta humilis* (Baker) T. Durand & Schinz	Zeaxanthin	Increased	[208]
N-containing compounds				
HT × eCO_2_	*Festuca arundinacea* Schreb.	Non-protein amino acids	Decreased	[200]
HT × LP	*Brassica oleracea* L.	Glucosinolates	Increased	[209]

Data are derived from the references shown. HT, higher temperature; aCO_2_, ambient CO_2_; eCO_2_, elevated CO_2_; DS, drought stress; LL, low light; HL, high light; UVB, ultraviolet-B radiation, BL, blue light; LP, longer photoperiod.

## Data Availability

Not applicable.
